# Unraveling Interactions between the Microbiome and the Host Immune System To Decipher Mechanisms of Disease

**DOI:** 10.1128/mSystems.00183-17

**Published:** 2018-03-06

**Authors:** Catherine A. Lozupone

**Affiliations:** a Division of Biomedical Informatics and Personalized Medicine, Department of Medicine, University of Colorado, Denver, Aurora, Colorado, USA

**Keywords:** graft-versus-host disease, T regulatory cell, capsular polysaccharide, gut microbiome, host-microbiome interaction, human immunodeficiency virus, multi-omic

## Abstract

In recent years, there has been a deluge of papers linking altered microbiome compositions to a myriad of diseases. Mechanistic insight into microbial drivers of disease phenotypes is essential for translation to novel therapies. A key mechanism by which microbes influence health is immune modulation by components of their capsule and cell envelope and their metabolites. A major research focus of my laboratory is to gain mechanistic insight into which microbes modulate host immunity generally and in the context of disease. Using 16S rRNA-targeted sequencing, we have established associations between gut microbiome composition and immune-modulated disease phenotypes in diseases such as graft-versus-host disease in cancer patients undergoing stem cell transplantation. By integrating omics and computational approaches with laboratory experiments, we have expanded knowledge of mechanisms used by host-associated microbes to dampen inflammatory responses. This work has promise for development of novel microbiome-targeted therapeutics.

## Abstract (precis)

In recent years, there has been a deluge of papers linking altered microbiome compositions to a myriad of diseases. Mechanistic insight into microbial drivers of disease phenotypes is essential for translation to novel therapies.

## PERSPECTIVE

Many diseases that plague our population share the common basis of a dysregulated immune system, including those characterized by a hyperactive immune response (e.g., asthma, allergy, and inflammatory bowel diseases) or by an ineffective immune response (e.g., susceptibility to opportunistic infections of the lung or gut or other sites) and those for which chronic low-grade inflammation is a potential contributing factor (e.g., obesity, depression, and dementia). Since our microbial inhabitants are in constant communication with the immune system on every surface of our body and are important for training the immune system in early life (1), understanding which microbes modulate immunity, and by what mechanism, has extreme broad implications.

## LOCAL VERSUS SYSTEMIC INFLUENCE OF MICROBES ON IMMUNE PHENOTYPES

It is well established that microbes locally influence immune status at the sites that they occupy (2). However, increasing evidence indicates that microbes can also influence peripheral immune cell populations, providing a mechanism by which microbes might influence disease pathology at distal sites (3). We found support for the idea of gut microbiome influence on peripheral immunity in a recent investigation of patients undergoing allogeneic bone marrow transplantation (aBMT) and their stem cell donors (4). In aBMT, immune cells from blood or bone marrow of a donor are transplanted into a person with a blood disorder, usually cancer. Optimally, the grafted effector cells mount an immune response to the cancer and the grafted stem cells reconstitute the immune system. An unfortunate complication is that the donor’s immune cells often attack microbially colonized surfaces of the patient, including the gut and skin, causing graft-versus-host disease (GVHD). Interestingly, we found that transplantation performed with immune cells from donors with relatively low gut microbiome diversity conferred increased risk for the development of gut GVHD (4). This observation has high translational potential; use of gut microbiome diversity as a screening criterion for donors and/or interventions to increase the gut microbiome diversity of preferred donors such as HLA-matched siblings may reduce risk of GVHD. This finding is also of great general interest because it supports the idea of a relationship between gut microbes and peripheral immunity. The therapeutic potential of understanding these relationships highlights the importance of elucidating the driving mechanisms.

## DISCOVERY PLATFORMS FOR IDENTIFYING NOVEL MECHANISMS OF IMMUNE INTERACTION

Microbes can modulate immunity through their cell envelope or capsular components (2) and also through small molecules produced during metabolism. For instance, the bacterial fermentation product butyrate can promote CD4^+^ T cell differentiation into proinflammatory or anti-inflammatory (Treg) phenotypes depending on the concentration and on which costimulatory factors are present (5). Also, indoxyl 3-sulfate, a derivative of indole which is produced by commensal microbes from tryptophan, has immune-regulatory functions that may confer protection from GVHD (6). In investigating microbe/immune interactions of importance in disease cohorts, one can investigate correlations between those molecules already known to mediate immune interactions and the presence or expression of the genes that encode them. However, we have probably only scratched the surface in identifying the variety of microbially produced components and metabolites that modulate immunity.

Use of integrated omic technologies, including shotgun metagenomics, metatranscriptomics, and metabolomics, to more deeply characterize phenotypes of both the microbiome and the host has the potential to generate hypotheses regarding novel mechanisms of immune modulation of importance in disease contexts. Challenges in these data-rich methods exist in differentiating signal from noise when so many statistical tests are being carried out, as well as in the analysis and interpretation of results. For instance, interpreting the potential implications of correlations between organisms, genes, or transcripts (as assessed with shotgun metagenomics or metatranscriptomics) and immune phenotypes is hampered by a poor understanding of what many of these organisms, genes, and transcripts actually do (7). Furthermore, in measuring the plasma metabolome by the use of untargeted liquid chromatography and mass spectrometry (LC/MS), the detected molecules represent a complex milieu consisting of small molecules produced by the host and by the microbiome as well as those that come from the environment, many of which cannot even be assigned a name. We have been using metabolic networks in KEGG (8) to identify which metabolites in our LC/MS data might have been produced by the microbiome. This approach is useful for metabolites that are a part of well-understood pathways but is hindered by a lack of this information for most small molecules (7). Thus, integrated multi-omic analysis, though promising, is only the first step toward mechanistic understanding. Functional exploration with *in vitro* assays, animal models, and genetic manipulation of bacteria is key for validating results.

## INVESTIGATING ELEMENTS OF THE BACTERIAL CAPSULE/CELL ENVELOPE AS A MECHANISM OF IMMUNE MODULATION

Levels of components of the bacterial cell envelope such as lipopolysaccharide (LPS) in blood have been linked with chronic inflammation in diseases such as HIV infection, indicating that therapies that discourage proinflammatory gut microbe growth and/or enhance intestinal barrier function have therapeutic potential (9). However, my research group has also been investigating microbial components that influence the other side of the immune-homeostasis balance, i.e., those that suppress inflammation. These may provide insight in designing therapies for diseases of chronic inflammation (10). Capsular zwitterionic polysaccharides (ZPSs), the best-studied of which is polysaccharide A (PSA) of the intestinal microbe *Bacteroides fragilis*, can suppress inflammation by stimulating CD4^+^ T cells to differentiate into an anti-inflammatory T regulatory (Treg) phenotype (11). We first became interested in ZPSs because CD4^+^ T cells are the primary target cell of HIV, and so we hypothesized that CD4^+^ T cell killing by HIV may lead to dysregulation of CD4^+^ T cell-mediated symbiotic relationships, thereby contributing to chronic inflammation in this population (10).

When we began our work, ZPSs of related immune-modulatory properties that could be produced by a few phylogenetically divergent bacteria were known, including PSA, SP1 of *Streptococcus pneumoniae*, and PSA2, a structural variant of PSA carried by a different strain of *B. fragilis* (11), but ZPSs were generally considered to be rare. However, by screening thousands of bacterial genome sequences, we predicted that strains of dozens of species from diverse bacterial phyla would have the necessary genes to make a molecule in the ZPS family (12). We verified that predicted ZPS producers had anti-inflammatory properties by comparing the abilities of phylogenetically integrated ZPS producers versus non-ZPS producers to induce interleukin-10 (IL-10) and Tregs in *in vitro* assays with peripheral blood mononuclear cells and also by evaluating protection in a mouse model of inflammatory disease. We further confirmed ZPS to be a driving factor of Treg and IL-10 induction in *in vitro* assays performed with *Bacteroides cellulosilyticus* by genetically disrupting ZPS operons (12). We are continuing to work in mouse models of inflammatory disease and in *in vitro* systems to establish whether any of these novel ZPS molecules or the microbes that produce them have therapeutic potential for diseases of chronic inflammation, including HIV infection. Understanding how to identify ZPS activity based on gene sequences will also allow us to investigate how the presence and expression of this function differ in various disease contexts using shotgun metagenomics and metatranscriptomic data sets, both generated in our laboratory and from the public domain.

## CONCLUSIONS

The microbiome field has come a long way in a short time. Although I am early in my scientific career, I can look back to how different things were just over a decade ago, when I was a Ph.D. student working with Rob Knight to develop a tool called UniFrac, which uses the information in phylogenetic trees to compute distances between microbial communities (10). When we first developed UniFrac, we had a “problem” that is funny to think about today; we wanted to couple UniFrac with multivariate statistics, but there were few data sets available at the time that included more than just a few different shallowly sequenced samples. Luckily, we were able to team up with Jeffrey Gordon for our first published UniFrac analysis, in a study that identified altered gut microbiome composition in a mouse model of genetic obesity using a total of 5,088 bacterial 16S rRNA gene sequences generated with Sanger sequencing from 19 mice (13). Although that total number of 5,088 sequences is paltry by today’s standards, it was one of the largest data sets of its time, and computational analysis of such a “large” data set was extremely challenging!

Although advances in next-generation sequencing and bioinformatics have been major drivers of progress in human microbiome research, there have also been other key advances that have enabled mechanistic insights. These include advances in our ability to culture diverse intestinal microbes and to genetically manipulate bacteria so that the effects of gain or loss of particular functions can be evaluated, the development of animal models such as gnotobiotic mice for establishing causality, and integration of other cutting-edge technologies such as metabolomics. Despite these gains, there are still many challenges, such as the high number of genes and metabolites of unknown function and of bacteria with poorly understood properties that evade cultivation and the lack of availability of tools to genetically manipulate most of those that we can culture. The most exciting and translational work integrates complex multi-omic and bioinformatics methods with confirmatory experimental work to establish a mechanistic link between microbes and disease. As a scientist early in my career with a particular expertise in computation in comparison to bench science, I have worked to establish strong collaborations with individuals with complementary expertise to perform integrated multidisciplinary research with the goal of developing effective microbiome-targeted therapeutics. With microbiome research accelerating at an impressive rate, one cannot help but be excited by the vast amounts of knowledge to be gained from the tiny world inside each of us.
